# Therapeutic potential of thymoquinone liposomes against the systemic infection of *Candida albicans* in diabetic mice

**DOI:** 10.1371/journal.pone.0208951

**Published:** 2018-12-27

**Authors:** Masood A. Khan, Yousef H. Aldebasi, Sultan A. Alsuhaibani, Mohammed A. AlSahli, Mohammad A. Alzohairy, Arif Khan, Hina Younus

**Affiliations:** 1 College of Applied Medical Sciences, Qassim University, Buraydah, Saudi Arabia; 2 Interdisciplinary Biotechnology Unit, Aligarh Muslim University, Aligarh, India; National Institute of Animal Biotechnology, INDIA

## Abstract

The present study was aimed to develop a liposomal formulation of thymoquinone (Lip-TQ) to treat *Candida albicans* infection in diabetic mice. Streptozotocin (STZ) was injected to induce hyperglycemia and on day 3 post STZ administration, mice were intravenously infected with *C*. *albicans*. Various doses (2, 5 and 10 mg/kg) of Free or Lip-TQ were administered in *C*. *albicans* infected diabetic mice. The effect of Lip-TQ was also determined on the organ indices, liver and kidney function parameters. Lip-TQ at a dose of 10 mg/kg significantly reduced the level of the blood glucose and alleviated the systemic *C*. *albicans* infection in diabetic mice. *C*. *albicans* infected diabetic mice treated with Lip-TQ at a dose of 10 mg/kg showed the survival rate of 70% as compared to that of 20% in the group treated with free TQ. The treatment with Lip-TQ resulted in the recovery of the organ indices, liver inflammation, kidney functioning and pancreas regeneration in diabetic mice. Moreover, TQ formulations also showed the direct therapeutic effect against candidiasis in the untreated or metformin-treated diabetic mice. Therefore, the findings of the present study support the use of Lip-TQ in the treatment of candidiasis in the diabetic patients.

## Introduction

*Candida albicans* is the most common opportunistic fungal pathogen that can infect the skin, oral cavity, gut and other mucous membranes in the immune-compromised subjects, including the patients undergoing extensive chemotherapy or organ transplantation, or infected with HIV are easy targets of *C*. *albicans* [1–3]. There are limited numbers of anti-fungal available and the current tendency of the emergence of antibiotic-resistant *C*. *albicans* is worrisome to clinicians and scientists [4–7]. The most common antifungal currently in use belongs to the polyene and azoles groups of drugs. But the use of polyene antifungals causes the acute hepatic and renal toxicity in the treated patients [8,9].

Diabetes mellitus is one of the most prevalent chronic conditions that causes morbidity due to its associated complications such as nephropathy, neuropathy, retinopathy and heart complications [10]. *C*. *albicans* is a predominant infectious agent that causes oral, esophageal and vaginal candidiasis in diabetic patients due to hyperglycemia [11]. Moreover, diabetic patients are found to be immunologically weak as compared to their normal counterparts [12]. The treatment of *C*. *albicans* in diabetic patients has been very challenging. It is necessary to regulate the blood glucose level in diabetic patients in order to make the anti-fungal therapy successful.

Thymoquinone (TQ), a major bioactive constituent of *Nigella sativa* seeds, is a multi-targeting molecule. It possesses a wide range of therapeutic properties including anti-oxidant, anti-inflammatory, anticancer, antimicrobial and anti-diabetic properties [13,14]. It shows anticancer and antitumor properties by regulating multiple molecular pathways [15]. TQ has shown anti-hyperglycemic effects in animal models [16]. An anti-diabetic effect of TQ was supposed to be mediated through the inhibition of nitric oxide (NO) and mitogen-activated protein kinases (MAPK) pathways [17].

The use of drug-delivery systems has been implemented to reduce the toxicity and increase the therapeutic index of polyene antifungal drugs [8, 9]. Thymoquinone possesses many beneficial therapeutic properties, but its use has been very limited owing to its poor solubility in an aqueous medium. In order to increase the solubility and bioavailability, it's important to incorporate TQ in the form of the liposomal formulation. The application of TQ has not been extensively investigated in the treatment of infectious diseases. In the present study, we intend to evaluate the therapeutic potential of the Lip-TQ against the systemic candidiasis in the diabetic mice.

## Materials and methods

### Materials

High quality Cholesterol and 1,2-dipalmitoyl-sn-glycero-3-phosphocholine (DPPC) were purchased from the Avanti Polar Lipids (Alabaster, Alabama, USA). Streptozotocin (STZ) and Thymoquinone were purchased from the Santa Cruz Biotechnology (Dallas, TX, USA). Sabouraud Dextrose Agar, Peptone and Yeast extract were purchased from the HiMedia Company (Mumbai, India). RPMI was purchased from the Sigma chemical company (St. Louis, MO, USA). Metformin hydrochloride was purchased from Sigma-Aldrich (St. Louis, MO, USA).

### C. albicans

*C*. *albicans* isolate was obtained from the Microbiology section of the King Fahad Hospital, Buraydah, Saudi Arabia.

### Mice

Female Swiss mice (10–12 weeks of age) were obtained from the animal house facility of the King Saud University, Riyadh, Saudi Arabia. All experiments involving the infection, bleeding, injection, as well as the sacrifice of mice were conducted following the guidelines and the approval of the animal ethics committee of the College of Applied Medical Sciences, Qassim University, Buraydah, Saudi Arabia. Mice were housed in the hygienic and pathogen-free conditions. The care of mice was taken by the well-trained and dedicated staff members. Mice were given an anesthetic injection of ketamine and xylazine mixture (90 mg/kg + 10 mg/kg) through an intra-peritoneal route before the infection with 5x10^5^ cells/mouse of *C*. *albicans*. Infected mice were observed two times a day for their mortality and morbidity for the whole duration of 40 days. To reduce the suffering, morbid mice (weight loss ≥25%) were immediately euthanized with CO_2_ and killed by cervical dislocation. None of the infected mice died during the observation period without being euthanized.

### Determination of minimum inhibitory concentration (MIC) of TQ

The activity of TQ against *C*. *albicans* isolate was determined by the broth dilution susceptibility method following the guidelines of Clinical and Laboratory Standards Institute (CLSI), Wayne, PA, USA [18]. A range of TQ concentrations (0.125 to 128 μg/ml) was used to determine the MIC against *C*. *albicans* in a 96-wells micro-titration plate. *C*. *albicans* was grown in YPD (1% yeast extract, 2% peptone and 2% dextrose). Cells were centrifuged and diluted in RPMI medium to give a final concentration of 5 x 10^3^ cells/ml. The plates were incubated at 37^0^ C for 48 hours. The optical density was measured at 530 nm and MIC was considered the lowest drug concentration that inhibited 90% growth as compared to the control.

### Preparation of TQ-loaded liposomes

A liposomal formulation of TQ (Lip-TQ) was prepared by a conventional thin-film hydration method [8]. DPPC, cholesterol and TQ were dissolved separately in a mixture methanol and chloroform (in a ratio of 1:1 Vol/Vol). The ratio of TQ and lipids was kept at 1:10. All the ingredients were taken in a round bottom flask. The solvents were evaporated to form a thin lipid film that was hydrated with sterile normal saline. The formulation was sonicated for a brief time.

### Determination of the size of TQ liposomes

The size of liposomes was determined by using the Mastersizer from the Malvern Instruments Ltd (Malvern, United Kingdom) as described earlier [19]. Lipid vesicles were passed 10 times through a 100 nm size of the membrane using a mini—extruder device from the Avanti polar lipids (Alabaster, AL, USA).

### Determination of the entrapment efficiency of TQ in liposomes

The amount of an un-entrapped TQ was separated from the liposomal TQ by centrifuging the formulation at 14,000 rpm. The amount of the liposomal TQ in liposomes was determined by measuring the absorbance at 330 nm using UV-Visible Spectrophotometer as described earlier [20]. A small aliquot of 50 μl of the liposomal TQ was disrupted in DMSO and the quantity of the released TQ was determined using the standard curve. The entrapment efficiency of TQ was calculated by measuring the amount of entrapped TQ out of the total amount of TQ originally added to the lipids.

%TQentrapmentefficiency=(TQentrappedintheLiposomes/TotalamountofTQ)X100

### Streptozotacin-induced diabetes in mice

Streptozotocin was reconstituted in 25 mM sodium citrate buffer at pH 4. Mice were kept overnight for fasting before the intra-peritoneal injections of streptozotocin (STZ) at a dose of 50 mg/kg for consecutive four days as described earlier [21]. Control mice were injected with the same amount of citrate buffer. The blood glucose level was monitored in each mouse post 24 hours of the last injection of STZ. Mice with a fasting glucose level ≥ 200 mg/dl were included in the study.

### Infection of *C*. *albicans* in diabetic mice

*C*. *albicans* (KFHS 121) was grown in YPD medium at 37 ^0^C for 48 h. The cell suspension was centrifuged at 5000 g for 15 min at 4 ^0^C in a cooling centrifuge followed by washing with the normal saline. On day 3 post STZ injection, each mouse was infected with 5 x 10^5^ CFU of *C*. *albicans* through an intravenous route as described earlier [8].

### Treatment of *C*. *albicans* infected diabetic mice with free TQ or Lip-TQ

The efficacy of various doses of free or Lip-TQ (2, 5 and 10 mg/kg) was determined against *C*. *albicans* Swiss mice. TQ was intraperitoneally injected on days 1, 3, 5 and 7 post *C*. *albicans* infection. The level of the blood glucose in the untreated or TQ-treated mice was determined on day 4, 6, 8 post treatment. Mice were divided into eight groups and each group contained ten mice: (1) Normal saline, (2) Sham Liposomes, (3) Free TQ (1 mg/kg), (4) Lip-TQ (1 mg/kg), (5) Free TQ (2 mg/kg), (6) Lip-TQ (2 mg/kg), (7) Free TQ (5 mg/kg), (8) Lip-TQ (5 mg/kg), (9) Free TQ (10 mg/kg), (10) Lip-TQ (10 mg/kg). The morbidity and mortality of mice were observed for a period of 30 days after the infection.

### Assessment of the severity of *C*. *albicans* infection in mice

The activity of free TQ or Lip-TQ against *C*. *albicans* was assessed by the survival data and fungal load in the kidney of the treated mice. The survival of mice was observed for 30 days post infection. To assess the severity of infection, three mice from each group were sacrificed on day 4 post treatment and their kidney was taken out to determine fungal load [8]. An equal amount of the weighed portions of the kidney were homogenized in an equal volume of the sterile normal saline and an aliquot of the suspension was plated on SDA plate. The plates were incubated at 37 ^0^C for 48 hours. The colony forming units (CFUs) were counted and the fungal load was calculated by multiplying by the dilution factor.

### Determination of the effect of TQ on the organic indices of vital organs in the diabetic mice

On day 4 post-treatment, the vital organs such as kidney, pancreas and liver were taken from the mice belonging to various groups and were washed with the phosphate-buffered saline (PBS). The organic index was calculated as follows: (organ weight) / (body weight).

### Determination of liver and renal toxicity markers in *C*. *albicans* infected diabetic mice untreated or treated with TQ formulations

On day 4^th^ post treatment, the blood was drawn from the mice of various experimental groups and the levels of liver inflammation markers, AST (Aspartate transaminase) and ALT (Alanine transaminase), and the values of the renal functional parameters such as the serum creatinine and blood urea were measured as described earlier [20].

### The histological analysis of the pancreatic tissues

The pancreas was taken from the diabetic mice belonging to the untreated or TQ-treated groups. The pancreatic tissues were fixed in a 10% neutral-buffered formalin solution. The paraffin-embedded blocks were prepared and the serial sections of 5 μm thickness were cut followed by Hematoxylin and Eosin (H and E) staining as described earlier [20]. The slides were rinsed and mounted with DPX. The pathological observations were made by examining the stained slide under the light microscope at the magnification of 200 (Lieca, USA).

### Determination of the direct anti-Candida effect of TQ formulations in the untreated and metformin-treated diabetic mice

In order to confirm the direct therapeutic efficacy of Lip-TQ against *C*. *albicans* in diabetic mice, *Candida albicans*-infected diabetic mice were treated with metformin at a dose of 250 mg/kg for 4 days followed by treatment with free TQ or Lip-TQ at a dose of 10 mg/kg. On day 4 post treatment, the blood glucose level was determined in the untreated or metformin-treated mice. Untreated and metformin-treated mice were further divided into the following groups: (1) Untreated control, (2) Metformin treated, (3) Free TQ, (4) Lip-TQ, (5) Metformin + Free TQ, (6) Metformin + Lip-TQ. The efficacy of the treatment was assessed by survival rate of the untreated or treated mice.

### Statistical analyses

Survival of the mice was shown using Kaplan–Meier curve. Analysis of survival was done using Log-rank Chi square test. The data of the fungal load in kidney were analyzed by one-way ANOVA using GraphPad Prism software, version 5.0 (La Jolla, CA, USA).

## Results

### Susceptibility of *C*. *albicans* isolates to TQ

The MIC value of TQ determined by a broth dilution method was found to be 5 μg/ml.

### Characterization of the size of liposomes and entrapment efficiency of TQ

The size of TQ-loaded liposomes was determined by the master sizer as described in the methodology section. There was a single peak near 100 nm. The entrapment efficiency of TQ in liposomal formulation was found to be about 90%.

### Treatment with Lip-TQ showed greater efficacy, compared to free TQ, to reduce the blood glucose level in diabetic mice

**Fig 1:**
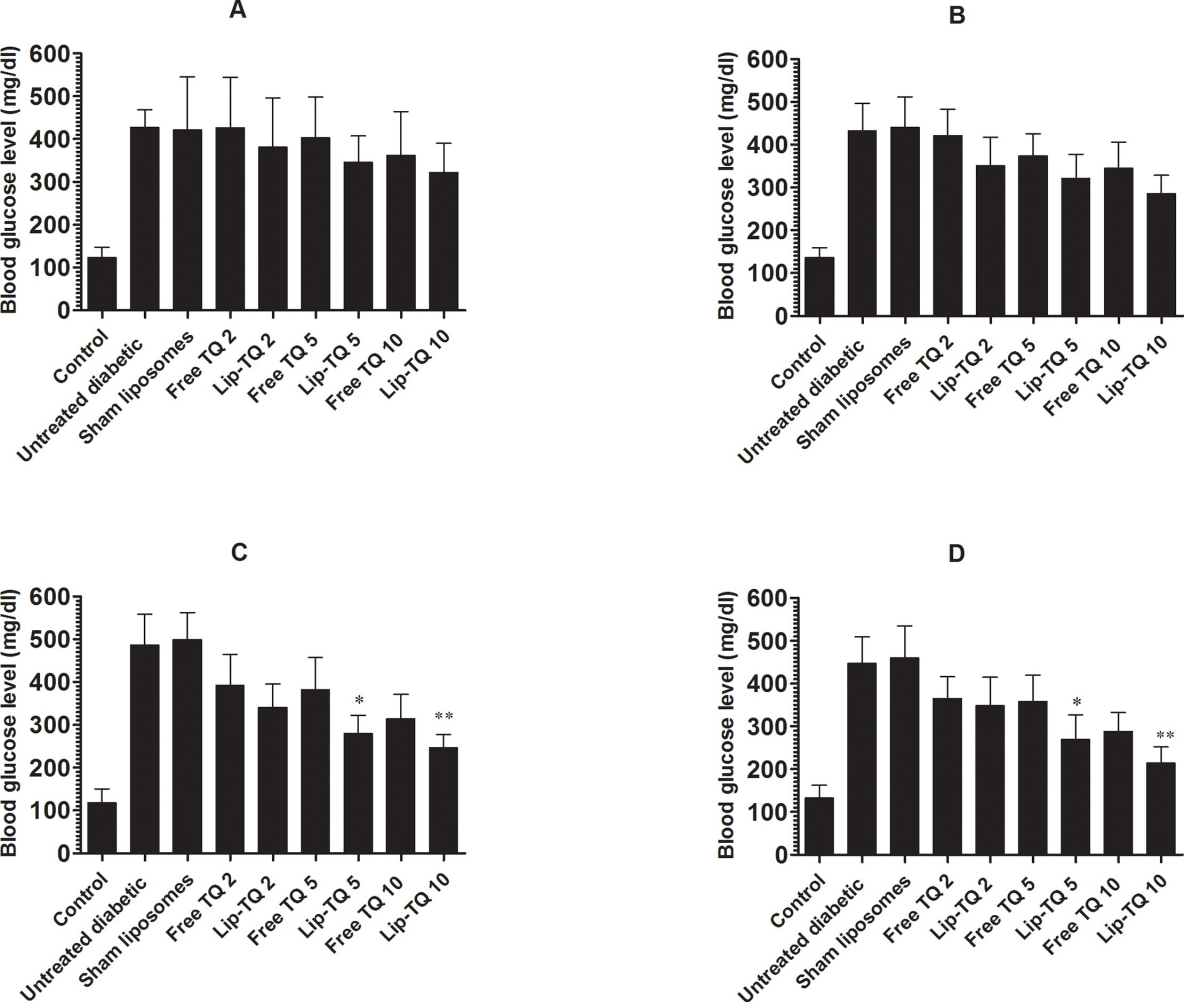
Treatment with the liposomal TQ results in the lowering of the blood glucose level in the diabetic mice. The level of the blood glucose in the diabetic mice post Lip-TQ treatment on day 4 **(A)**, day 6 **(B)**, day 8 **(C)** and day 10 **(D)**. On day 8 post-treatment, Diabetic mice vs Lip-TQ- 5mg/kg (P<0.05); Diabetic mice vs Lip-TQ- 10 mg/kg (P<0.01). On day 10, Diabetic mice vs Lip-TQ- 5mg/kg (P<0.05); Diabetic mice vs Lip-TQ- 10 mg/kg (P<0.01).

Due to poor solubility in an aqueous medium, free TQ does not show a substantial absorption and thus its activity is compromised. In order to increase *in vivo* action, a liposomal formulation of TQ was prepared and its anti-diabetic activity was assessed in mice. Lip-TQ showed greater efficacy in reducing the level of blood glucose as compared to free TQ at the same dose. Diabetic mice, the untreated group, showed much higher levels of the blood glucose, 427 ± 41 on day 4, 432 ± 64 on day 6, 486 ± 72 on day 8, 447 ± 31 on day 10 as compared to the blood glucose level of about 123 ± 24, 136 ± 23, 118 ± 32, 132 ± 30 in normal control mice on the respective days (Fig 1A). Treatment with a lower dose of TQ (2 mg/kg) did not significantly reduce the blood glucose levels over the period of treatment (Fig 1A, 1B, 1C and 1D). However, the treatment of the diabetic mice with Lip-TQ at higher doses (5 mg/kg and 10 mg/kg) significantly reduced the blood glucose level, particularly on days 8 and 10 post-treatment (Fig 1C and 1D). Free TQ at higher doses (5 mg/kg and 10 mg/kg) did reduce the level of blood glucose, but this reduction was not significant even on days 8 and 10 post-treatment (Fig 1C and 1D). Lip-TQ at a dose of 5 mg/kg reduced the blood glucose levels to 280 ± 42.5 on day 8, and 269 ± 52.5 on day 10 respectively (Fig 1C and 1D) (P<0.05). Whereas, free TQ at a dose of 5 mg/kg reduced the blood glucose levels to 382 ± 75 and 357.6 ± 62.5 on the same days (Fig 1C and 1D). Similarly, the diabetic mice in the group treated with Lip-TQ at a dose of 10 mg/kg have the blood glucose level of 246.6 ± 30.3 on day 8, and 214 ± 38 on day 10 respectively (Fig 1C and 1D) (P<0.01). On the other hand, the group of diabetic mice treated with free TQ at a dose of 10 mg/kg have the blood glucose level of 314 ± 57 on day 8, and 288 ± 44 on day 10 respectively (Fig 1C and 1D). Thus, Lip-TQ showed greater activity in reducing the blood glucose level in the diabetic mice.

### Treatment of Lip-TQ increases the survival and reduces the severity of *C*. *albicans* infection in diabetic mice

**Fig 2:**
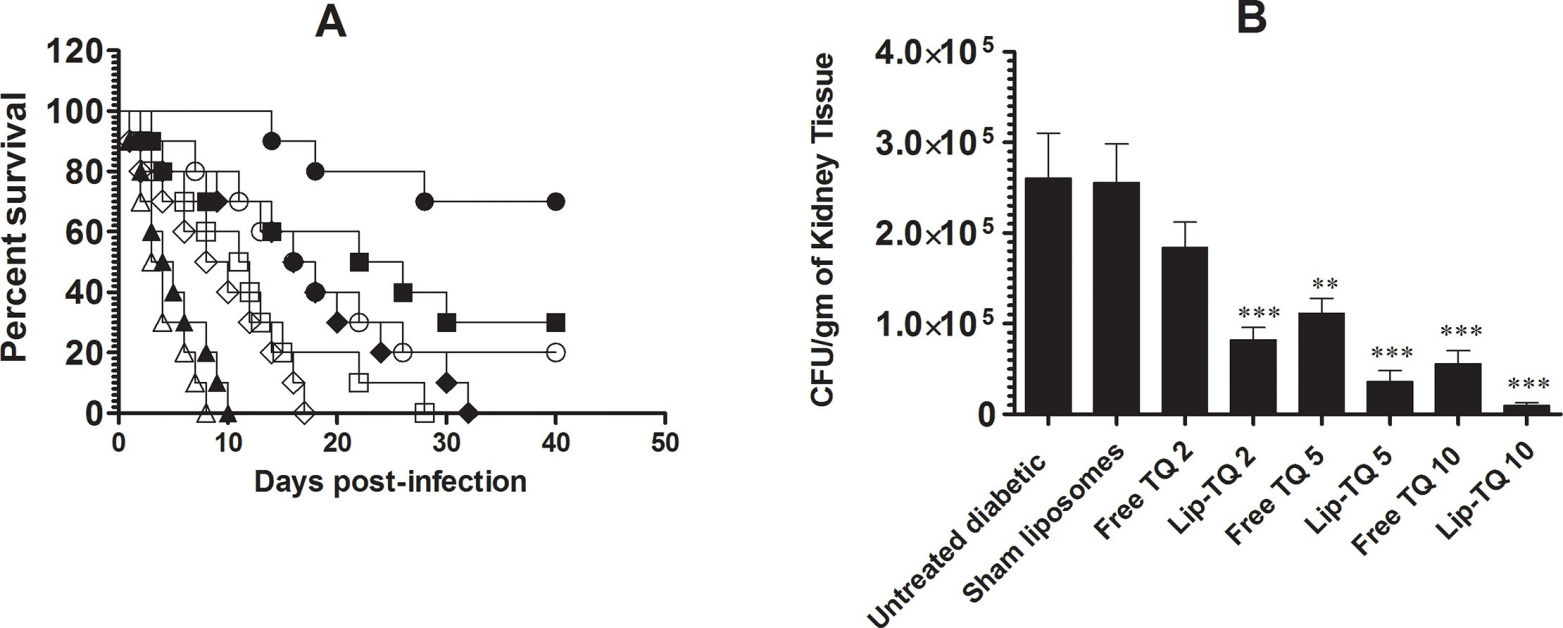
Treatment with Lip-TQ was effective against *C*. *albicans* infection in the diabetic mice. **(A)** The survival of *C*. *albicans* infected diabetic mice was observed for 40 days post treatment. Untreated diabetic (△), Sham Liposomes (▲), Free-TQ-2 mg/kg (◊), Lip-TQ-2 mg/kg (◆), Free-TQ-5 mg/kg (□), Lip-TQ-5 mg/kg (■), Free-TQ-10 mg/kg (○), Lip-TQ-10 mg/kg (●). Lip-TQ-10 mg/kg vs free TQ-10 mg/kg (P = 0.0136), Lip-TQ-5 mg/kg vs free TQ-5 mg/kg (P = 0.0356), **(B)** On day 4 post treatment, three mice from each group were sacrificed and their kidney were homogenized. The homogenates from an equally weighed kidney tissue were cultured to determine the fungal load. Values are expressed as mean ± SD from kidney from three different mice. A *P* value <0.05 was considered to be significant.

Lip-TQ was highly effective in eliminating *C*. *albicans* infection in the diabetic mice. All diabetic mice infected with *C*. *albicans*, in the untreated group, showed a median survival of 3.5 days and died by day 8 post-infection (Fig 2A). The mice in the group treated with Lip-TQ at a dose of 10 mg/kg showed 70% survival on day 40 post treatment as compared to 20% survival of mice in the group treated with free TQ at the same dose (P = 0.0136). *C*. *albicans* infected diabetic mice in the group treated with Lip-TQ at a dose of 5 mg/kg showed 30% survival, whereas all mice in the group treated with free TQ died before day 40 of observation (Fig 2) (P = 0.0356). Lip-TQ at a lower dose showed greater activity as compared to free TQ at the same or higher doses (Fig 2A). Mice in the group treated with Lip-TQ at a dose of 2 mg/kg showed median survival 17 days, as compared 11.5 days in the group of mice treated with free TQ at a dose of 5 mg/kg (Fig 2A).

The severity of infection in the diabetic mice was measured by determining the fungal burden in the kidneys of the mice untreated or treated with various formulations of TQ. Mice in the untreated group infected with *C*. *albicans* exhibited severe infection in their kidney. The CFUs of *C*. *albicans* in the kidney of the untreated mice was very high and found to be 260484 ± 49990 per gram of the tissue (Fig 2B). Mice in the group treated at a dose of 10 mg/kg of Lip-TQ showed the lowest fungal load (9364 ± 3460 CFUs) in the kidney tissues as compared to 55614 ± 14607 CFUs in the group of mice treated at the same dose of free TQ (Fig 2B). Similarly, the CFUs of *C*. *albicans* was found to be 36329 ± 11685 in the kidney of mice from the group treated with Lip-TQ at a dose of 5 mg/kg as compared to 111542 ± 16765 in the kidney of mice treated with free TQ at the same dose (Fig 2B). It shows that treatment with Lip-TQ was more effective in eliminating *C*. *albicans* from the tissues of the infected mice.

### Treatment with Lip-TQ improved the organ indices in *C*. *albicans* infected diabetic mice

**Fig 3:**
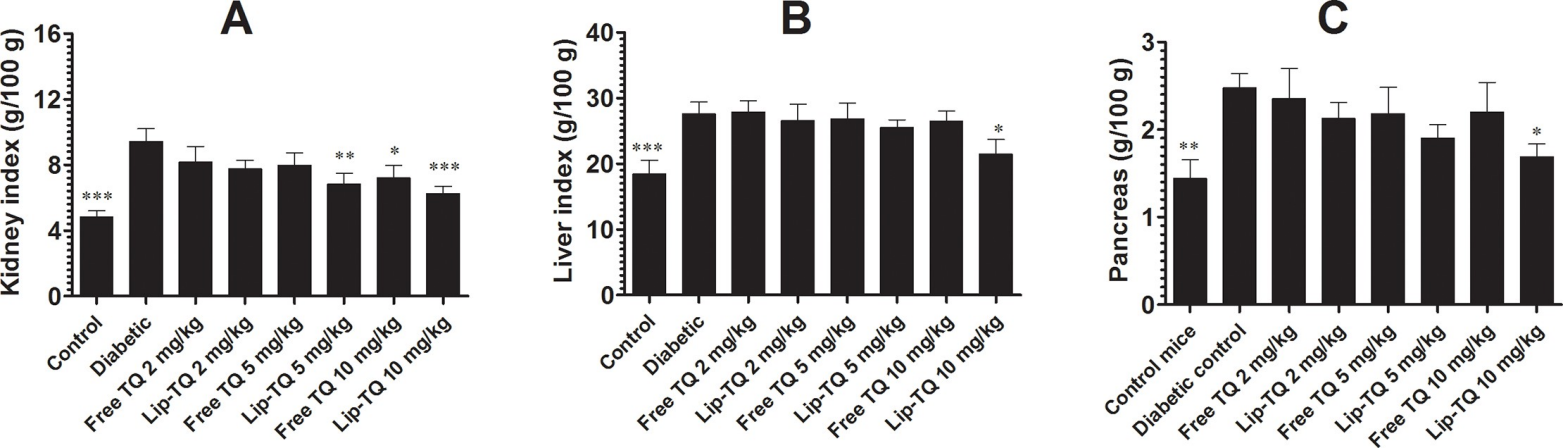
Treatment with Lip-TQ improves the organ index in *C*. *albicans* infected diabetic mice. On day 4 post-treatment, the kidney, pancreas and liver were taken from the mice of various groups and were washed in PBS. The organic index of **(A)** kidney, **(B)** liver and **(C)** pancreas, was calculated as follows: organ weight / body weight. Data are expressed as mean ± SD from organs three different mice. A *P* value <0.05 was considered to be significant.

The indices of vital organs were assessed in mice from groups untreated or treated with TQ formulations. *C*. *albicans* infected diabetic mice treated with Lip-TQ showed the enhanced improvement in their organ indices as compared to the mice from the untreated or free TQ treated groups. Diabetic mice infected with *C*. *albicans* showed kidney index of 9.43 ± 0.77 as compared to 4.82 ± 0.38 of control mice (Fig 3). The group of *C*. *albicans* infected diabetic mice treated with Lip-TQ at a dose of 10 mg/kg exhibited the kidney index of 6.26 ± 0.44 (P<0.001). Like to kidney index, the indices of liver and pancreas were increased from 18.48 ± 2.1 to 27.6 ± 1.8 and 1.44 ± 0.22 to 2.47 ± 0.17 respectively. Treatment with Lip-TQ at a dose of 10 mg/kg reduced the liver index from 27.6 ± 1.8 to 23.2± 1.56, and pancreas index from 2.47 ± 0.17 to 1.70 ± 0.14 (P<0.05). This suggests that treatment with Lip-TQ was effective in improving the organ indices in the diabetic mice infected with *C*. *albicans*.

### Treatment with Lip-TQ reduces the liver inflammation in *C*. *albicans* infected diabetic mice

**Fig 4:**
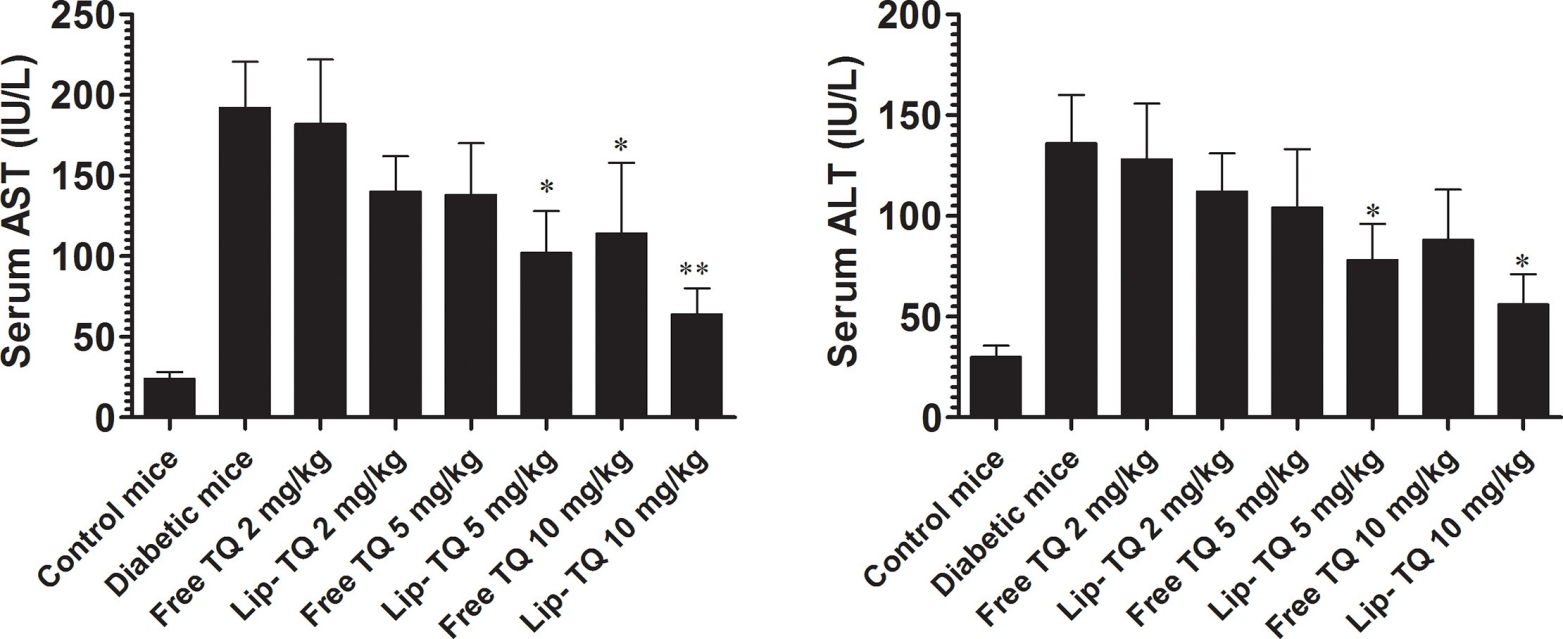
Treatment with Lip-TQ improves the liver inflammation in *C*. *albicans* infected diabetic mice. Free or Lip-TQ at the doses of 5 and 10 mg/kg were administered to treat *C*. *albicans* infected diabetic mice as described in the methodology section. On day 4^th^ post treatment, the blood was drawn from the mice to measure the levels of **(A)** AST, **(B)** ALT. Data are expressed as mean ± SD. A *P* value <0.05 was considered to be significant.

The administration of streptozotocin has been associated with inflammation and malfunction of vital organs [22]. The group of *C*. *albicans* infected diabetic mice showed significantly higher levels of AST (192 ± 28) and ALT (136 ± 24) as compared to the respective values of AST and ALT, 24 ± 4.2 and 30 ± 5.6, in the mice from the control group (Fig 4A and 4B). The groups of *C*. *albicans* infected mice treated with free TQ at a dose of 10 mg/kg exhibited the AST value of 114 ± 44 as compared to 192 ± 28 in the untreated group (P<0.05), whereas the mice in the group treated at the same dose of Lip-TQ showed a higher reduction in the level of AST (64 ± 16) (P<0.001). Unlike to the treatment with free TQ at a dose of 5 mg/kg that insignificantly reduced the level of AST from 192 ± 28 to 138 ± 32, treatment with Lip-TQ at the same dose significantly reduced the AST level from 192 ± 28 to 102 ± 26 (P<0.05).

The level of ALT, another marker of liver inflammation, was also measured in the mice that belong to various experimental groups. *C*. *albicans* infected diabetic mice showed a significantly greater level of ALT, 136 ± 24, as compared to 30 ± 5.6 in the normal control group (P<0.001). The group of *C*. *albicans* infected diabetic mice treated with free TQ at a dose of 10 mg/kg exhibited an ALT value of 88 ± 25 (P>0.05), whereas Lip-TQ at the same dose significantly reduced an ALT value to 56 ± 15 (P<0.05). Moreover, treatment with Lip-TQ at a dose of 5 mg/kg significantly decreased an ALT level to 78 ±18 (P<0.05). Thus, the results of the present study demonstrated that Lip-TQ, not free TQ, was more effective in reducing the inflammation of the liver in the diabetic mice.

### Treatment with Lip-TQ improved the renal function in the *C*. *albicans* infected diabetic mice

**Fig 5:**
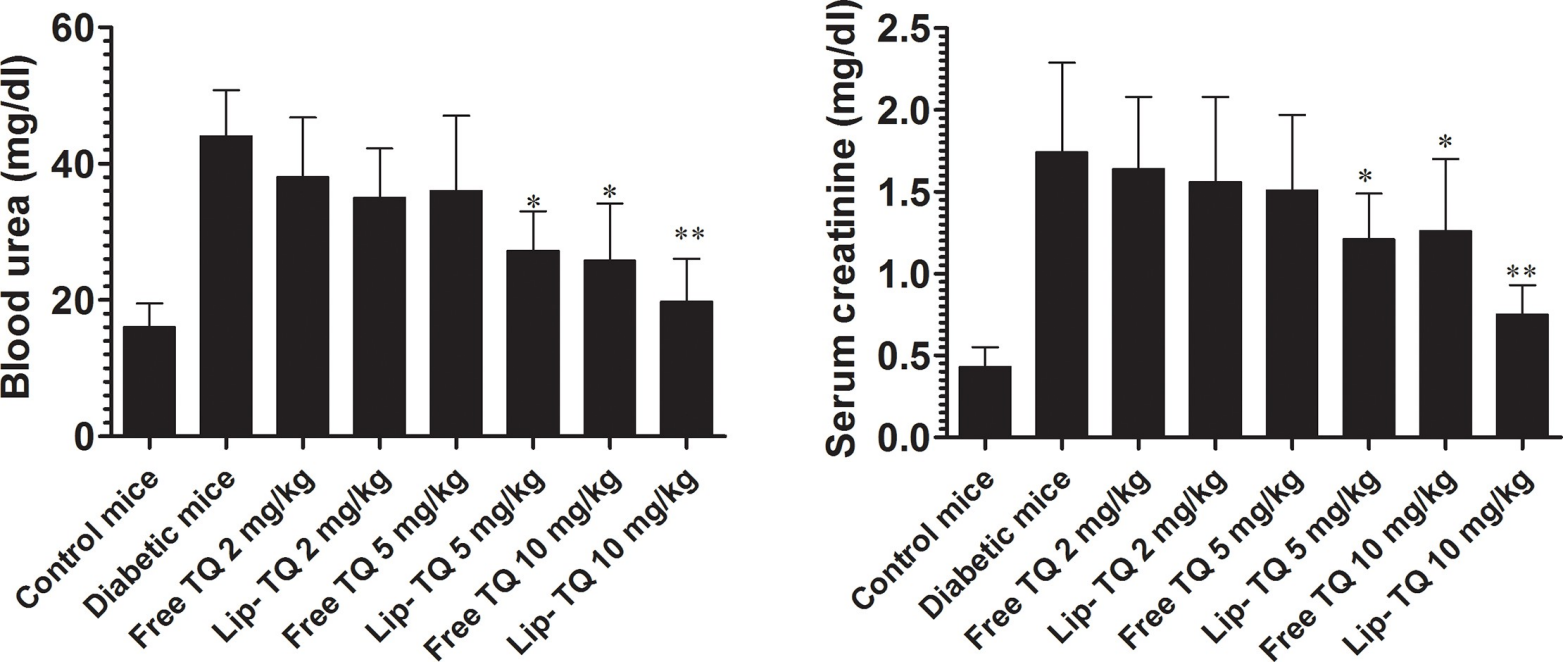
Treatment with Lip-TQ improves the functioning of the kidneys in *C*. *albicans* infected diabetic mice. On day 4^th^ post treatment, the blood was drawn from the mice of various groups to measure the levels of **(A)** blood urea, **(B)** serum creatinine. Data are expressed as mean ± SD. A *P* value <0.05 was considered to be significant.

The group of diabetic mice infected with *C*. *albicans* showed higher levels of serum creatinine and blood urea that were significantly reversed after treatment with Lip-TQ at a dose of 10 mg/kg (Fig 5A). The level of serum creatinine was increased to 1.74 ± 0.55 in the diabetic mice as compared to 0.42 ± 0.12 in the control mice (P<0.01). Treatment with Lip-TQ at a dose of 10 mg/kg significantly decreased the serum creatinine level in the diabetic mice from 1.74 ± 0.55 to 0.75 ± 0.18 (P<0.01), whereas treatment with free TQ at the same dose decreased creatinine level to 1.26 ± 0.44 (P<0.05). Moreover, treatment with Lip-TQ at a dose of 5 mg/kg was effectively able to reduce the creatinine level to 1.21 ± 0.28 (P<0.05).

Like to serum creatinine, the level of blood urea was also increased to 44 ± 6.8 in the diabetic mice as compared to 16 ± 3.5 in control mice (Fig 5B). The group of diabetic mice treated with Lip-TQ at a dose of 10 mg/kg showed the blood urea level of 19.8 ± 6.30 as compared to 44 ± 6.8 in the untreated diabetic mice (P<0.01), whereas the group of diabetic mice treated with free TQ at the same dose had 25.8 ± 8.4 of the urea level (P<0.05). However, treatment with Lip-TQ at a lower dose (5 mg/kg) effectively reduced the level of urea to 27.2 ± 5.8 (P<0.05). In contrast, free TQ at a dose of 5 mg/kg did not significantly reduce the blood urea level (P>0.05). This finding suggested that Lip-TQ was more effective than free TQ to normalize the kidney functioning in the diabetic mice.

### Treatment with Lip-TQ protected the integration of the pancreatic tissues in diabetic mice

**Fig 6:**
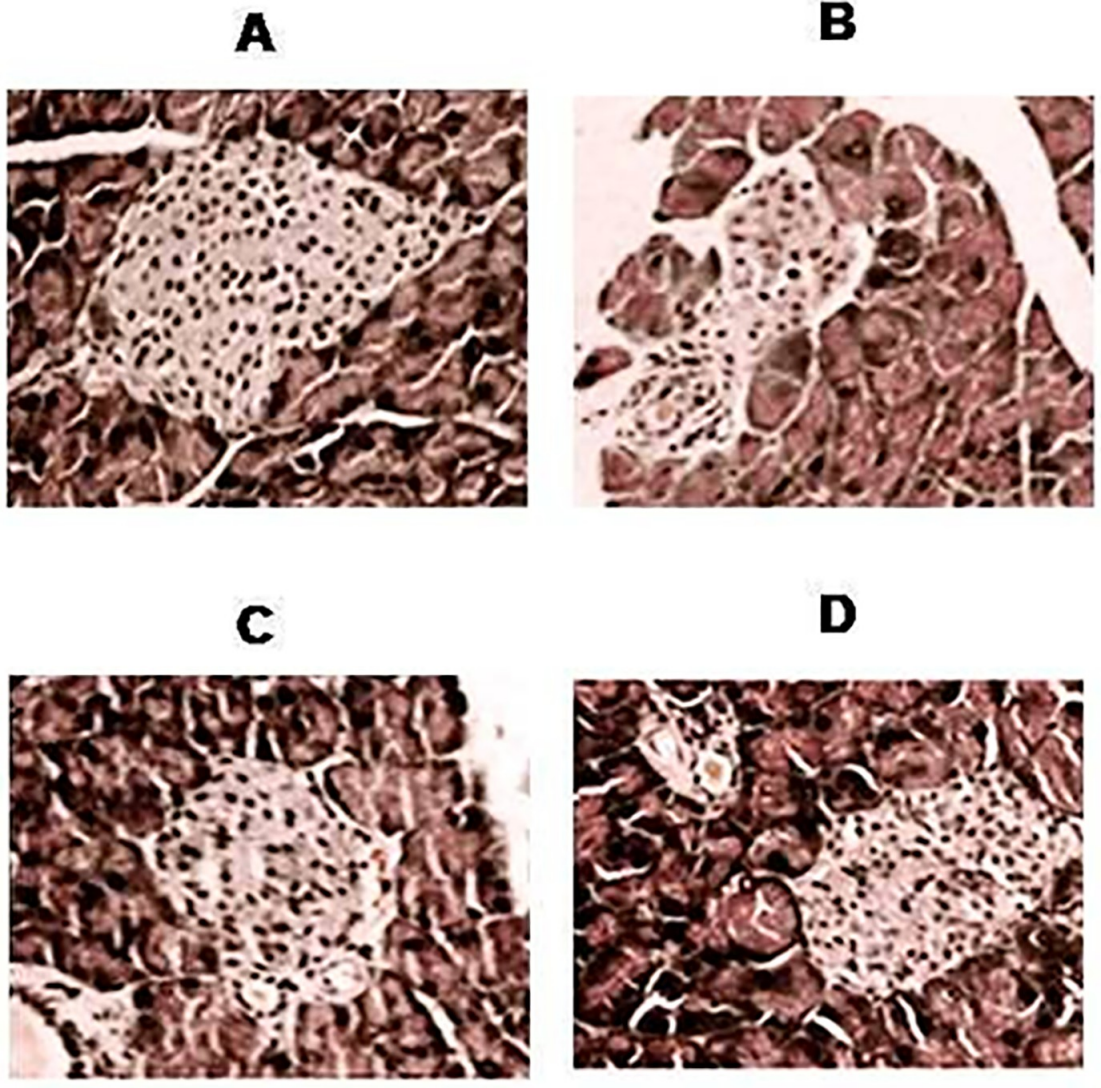
Treatment with Lip-TQ repairs the STZ-induced degeneration of pancreatic tissues. On day 4^th^ post treatment, the pancreas was taken from the mice of various groups. The pancreatic tissues were processed for the histological analysis and observed at 200 X magnification after H & E staining. **(A)** Normal control, **(B)** Diabetic, **(C)** Free TQ-10 mg/kg, **(D)** Lip-TQ-10 mg/kg.

In order to observe the alterations at the tissue level, the pancreas taken from the untreated or free TQ or Lip-TQ treated diabetic mice were processed for the histopathological studies. Pancreatic tissues from the untreated diabetic mice showed severe disintegration, including hypochromatosis, the infiltration of lymphocytes and the vanishing of cell borders in the pancreatic islets as compared to pancreatic tissues from the normal mice (Fig 6A and 6B). For the treatment with Lip-TQ or free TQ at a dose of 10 mg/kg was effective in ameliorating the hyperglycemia in STZ-injected mice, the pancreatic tissues of the mice from the groups treated with Lip-TQ or free TQ (10 mg/kg) was included in the histopathological studies. The treatment with Lip-TQ at a dose of 10 mg/kg reversed STZ-induced toxic manifestations in the diabetic mice (Fig 6C and 6D). The pancreas from the group of diabetic mice treated with Lip-TQ at a dose of 10 mg/kg showed a lower infiltration of lymphocytes, more integrated structure of pancreatic islets and re-appearance of cell borders as compared to the tissues from the untreated or free TQ treated mice. These results revealed that treatment of diabetic mice with Lip-TQ repaired the damaged tissues and recovered the cellular integrity in the pancreas.

### Treatment with Lip-TQ shows the direct therapeutic effect against *C*. *albicans* in the untreated or metformin-treated diabetic mice

**Fig 7:**
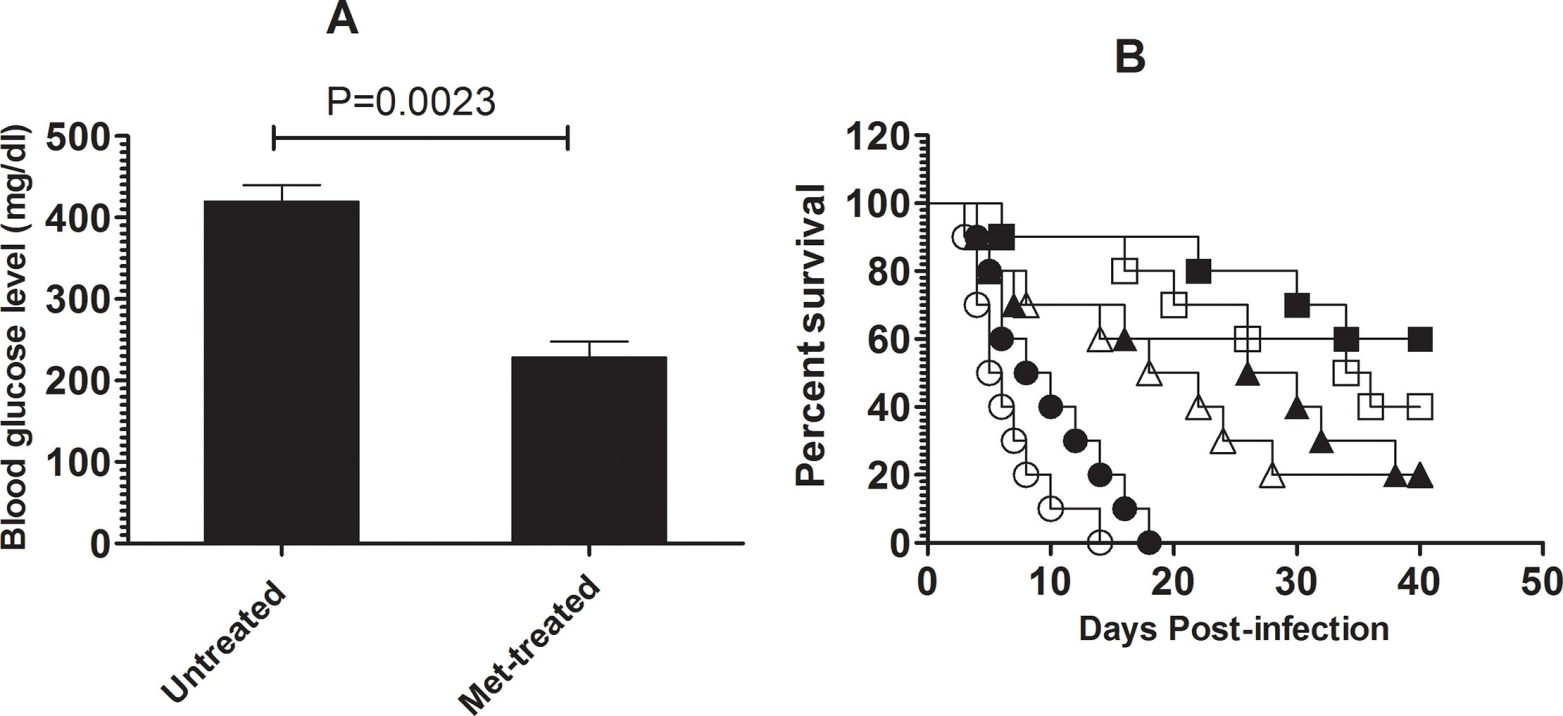
Thymoquinone formulations showed anti-candida activity in the untreated or metformin-treated diabetic mice. **(A)** Treatment of the diabetic mice with metformin resulted in the significant reduction of the blood glucose level (P = 0.0023). **(B)** The survival of *C*. *albicans* infected diabetic mice untreated or treated with TQ formulations were observed for 40 days post treatment. Untreated diabetic (○), Metformin-treated diabetic (●), Free TQ (△), Met + Free TQ (▲), Lip-TQ (□), Met + Lip-TQ (■).

The direct therapeutic effect of TQ was investigated in the untreated or metformin-treated diabetic mice. Treatment with metformin significantly reduced the level of the blood glucose in the diabetic mice (P = 0.0023) (Fig 7A). The blood glucose level in the untreated diabetic mice was found to be 419.7 ± 20.12, whereas metformin-treated diabetic mice showed the blood glucose level of 228.7 ± 19.06 (Fig 7A).

Moreover, the anti-candia effect of free TQ or Lip-TQ also investigated in both metformin-treated and untreated mice. Lip-TQ, at a dose of 10 mg/kg, was the most effective against *C*. *albicans* infection in both untreated and metformin-treated diabetic mice with a survival rate of 40% and 60%, respectively (Fig 7B), whereas treatment of the diabetic mice with free TQ resulted 30% and 40% survival rate respectively (Fig 7B). Interestingly, treatment with Lip-TQ was more effective in metformin-treated mice as compared to the untreated mice, but this effect was found to be insignificant (P = 0.3961). These results showed that therapy with TQ formulations demonstrated the direct anti-candida activity in both untreated and metformin-treated diabetic mice.

## Discussion

The results of the present study demonstrated that a liposomal formulation of TQ reduced the severity of hyperglycemia and effectively eliminated *C*. *albicans* infection in diabetic mice. Moreover, Lip-TQ also showed the direct therapeutic effect against *C*. *albicans* in the untreated or metformin-treated diabetic mice. Hyperglycemia has been associated with the increased frequency of the fungal infections, particularly in the oral cavity, gastrointestinal tract, skin, foot and vagina [23]. *C*. *albicans* is the most common fungal pathogen in diabetic patients up to 80% of prevalence with a high degree of morbidity due to oral candidiasis [24,25]. Moreover, the growth of *C*. *albicans* in the form of the bio-film increases the virulence of the pathogen [26].

Despite of possessing many beneficial therapeutic properties, the use of TQ has been very limited in the clinical setting owing to its lipophilic property, sensitivity to light, temperature and pH. For many years, the liposomes have been considered a superb drug delivery system because they can efficiently be loaded with the lipophilic drugs that otherwise have poor absorption. The lipophilic drugs, including TQ can be considered very effective when used as a liposomal formulation. The delivery of TQ in nano-particles has been shown its implications in the treatment of cancer [27]. In another study, TQ-loaded liposomes inhibited the proliferation of breast cancer MCF-7 and T47D cell lines [28]. Moreover, TQ-loaded gold niosomes showed the ability to overcome drug-resistant MCF7 breast cancer cell line [29]. The increased efficacy of liposomal TQ, as compared to free TQ, is also supported by the results of the present study that showed the enhanced anti-hyperglycemic effect of Lip-TQ in the diabetic mice. Lip-TQ was significantly more effective than free TQ to lower down the blood glucose level at the comparative doses (Fig 1C and 1D). Interestingly, the treatment with Lip-TQ at a dose of 5 mg/kg showed superior anti-hyperglycemic activity to that of free TQ at a dose of 10 mg/kg (Fig 1C and 1D). The greater anti-hyperglycemic activity of Lip-TQ can be attributed to its increased solubility and bioavailability in the liposomal formulation.

TQ has shown its activity against a variety of pathogens, including bacteria and fungi [30–32]. In the last two decades, a number of natural products have been reported to possess an anti-Candida activity. *N*. *sativa* oil and its extract have shown their activities against almost all *Candida spp*, including *C*. *albicans*, *C*. *glabrata*, *C*. *krusei* and *C*. *parapsilosis* [33]. In order to control *C*. *albicans* infection in the diabetic patients, it is important to bring the blood glucose down to the normal level. Until the blood glucose is brought to the normal level, the severity of *C*. *albicans* cannot be effectively controlled. In the present study, STZ was injected in mice to induce hyperglycemia and the diabetic mice were exposed to the systemic infection of *C*. *albicans*. The results of the present study demonstrated that *C*. *albicans* causes severe infection in the diabetic mice. The diabetic mice infected with *C*. *albicans* showed the highest survival rate about 70% after the treatment with Lip-TQ at a dose of 10 mg/kg, whereas the mice in the group treated with free TQ at the same dose showed only 20% survival on day 40 post-treatment. Interestingly, Lip-TQ at a dose of 5 mg/kg showed 30% survival. Thus the treatment with Lip-TQ was more effective not only in regulating the blood glucose level, but also eliminated *C*. *albicans* from the diabetic mice.

The administration of STZ induces the inflammation that results in an increase of organ indices as shown by the results of the present study (Fig 3). We have earlier shown that treatment with TQ reduces the liver inflammation in cyclophosphamide-injected mice [20]. The results of the present study demonstrated that STZ-injected mice showed the greater index of the kidney, liver and pancreas that were reversed in the mice treated with Lip-TQ. Thus, Lip-TQ showed superior anti-inflammatory activity as compared to free TQ at the same dose.

A long term hyperglycemia causes the impairment of the organ functioning and the natural products have ability to reverse the alterations in the diabetic mice [34,35]. In the present study, the diabetic mice infected with *C*. *albicans* showed the highest levels of liver inflammation markers such as AST and ALT in their blood. TQ possesses very strong anti-inflammatory and hepatoprotective activities [20]. The results of the present study demonstrated that mice in the group treated with Lip-TQ showed the highest reduction in AST and ALT levels as compared to the untreated diabetic mice. The kidney is one of the most affected organs in the diabetes. It increases the kidney size and glomerular volume, leading to the diabetic retinopathy. Hyperglycemia also induces the generation of the oxidative stress and advanced glycation end products (AGEs) that are important players in the kidney malfunctioning. In earlier studies, we have shown that TQ possesses anti-oxidant and anti-glycating activities [36]. Moreover, treatment with Lip-TQ also recovered the depleted activity of the anti-oxidant enzymes in mice [20]. The results of the present study demonstrated that diabetic mice showed higher levels of serum creatinine and blood urea. Treatment of diabetic mice with Lip-TQ significantly lowered the increased levels, serum creatinine and blood urea. The nephro-protective effect of TQ may be attributed to its anti-oxidant and anti-glycating activities.

The administration of streptozotocin causes the cytotoxicity to β-cells of the pancreas by increasing the levels of free radicals. Certain phytochemicals can repair the STZ-induced damage of the pancreatic β-cells by scavenging the free radicals [37]. The results of the present study demonstrated that STZ caused the damage of pancreatic tissues and the treatment of the diabetic mice with Lip-TQ, at a dose of 10 mg/kg, remarkably protected the degeneration of the β-cells of the islets of Langerhans in the pancreas. This result is in accordance with the earlier studies that showed the protective effect of *N*. *sativa* against STZ-induced damage of the pancreas [38]. Above mentioned findings showed the anti-diabetic and anti-Candida activity of free and Lip-TQ as well. The direct therapeutic effect of TQ formulations was also determined in the untreated or metformin-treated mice. Lip-TQ showed the highest efficacy against *C*. *albicans* in both untreated and metformin-treated diabetic mice. This confirms that anti-Candida activity of TQ is not dependent on its anti-diabetic activity. The minor reduction in an anti-candida activity of TQ formulations in the untreated diabetic mice as compared to metformin-treated mice is owing to the more favorable conditions of *C*. *albicans* proliferations in the former group.

The findings of the present study substantiate the use of Lip-TQ in the treatment of candidiasis in diabetic patients. TQ is a very safe drug because of its high LD_50_ values i.e. 104.7 mg/kg through intra-peritoneal route and 870.9 mg/kg through the oral route. The formulation of TQ in the liposomal form further decreases its toxicity and increases activity. Thus liposomal formulations of TQ may be considered to be used in the clinical setting, particularly in diabetic patients or immunocompromised persons suffering from candidiasis [39].

## Conclusions

**Fig 8:**
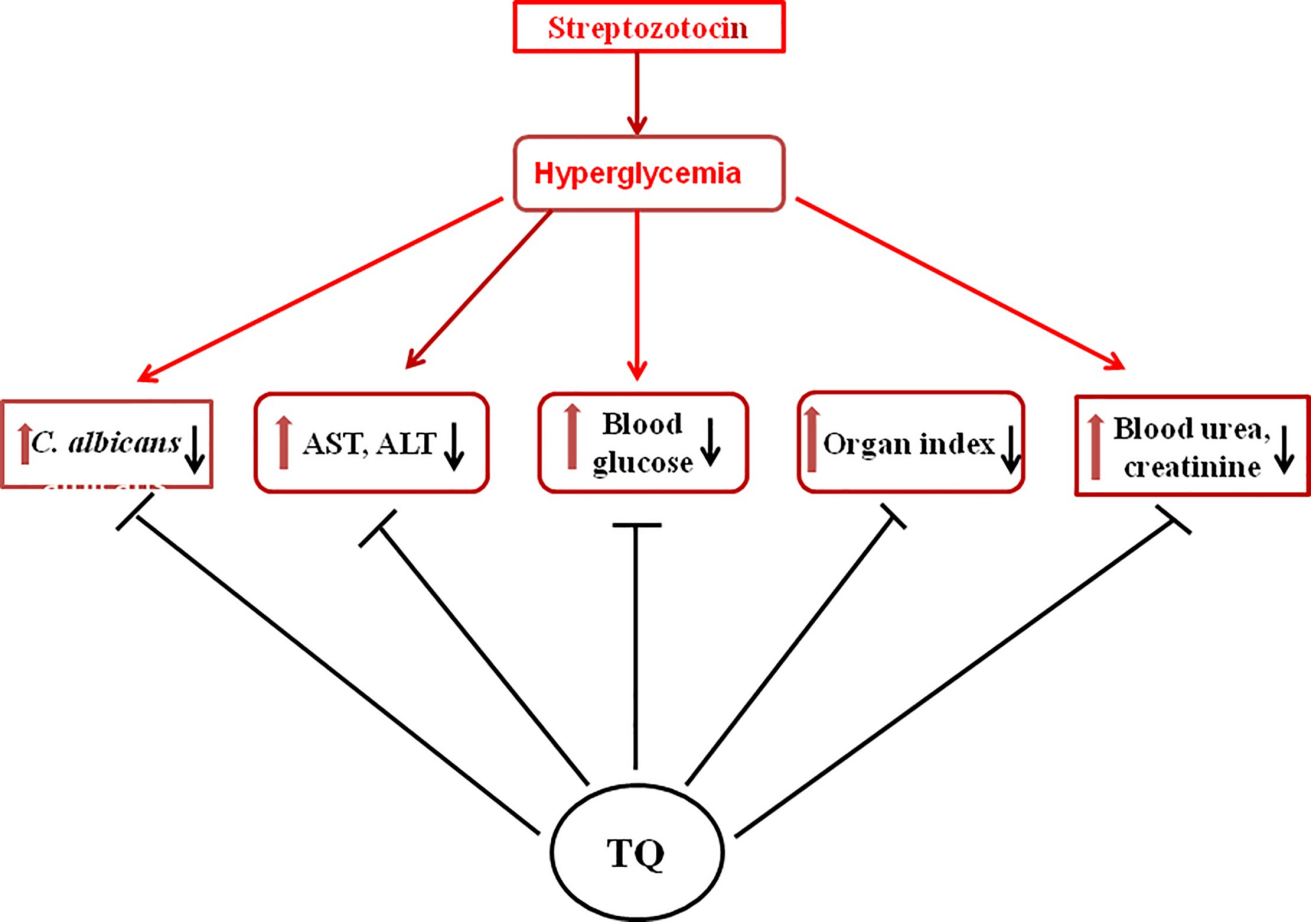
Multi-targeting effects of TQ against *C*. *albicans* infection and diabetes-associated complications.

In the present study, it was observed that treatment with Lip- TQ not only eliminated *C*. *albicans* infection of the diabetic mice, but also reduced the severity of diabetes-associated complications (Fig 8). The administration of Lip-TQ resulted in the reduction of the organ indices in the untreated or treated mice. Moreover, treatment with Lip-TQ also reduced the levels of the hepatic inflammation markers and repaired the kidney functioning in the diabetic mice. Interestingly, the structural integrity was restored in the β-cells of the pancreas of the mice treated with Lip-TQ. The outcomes of the present study suggest that the use of Lip-TQ may be beneficial to treat the diabetic patients suffering of candidiasis.
